# *ACTN3* rs1815739 and *BDNF* rs6265 Polymorphisms May Not Be Associated with Handgrip Strength in Elite Wrestlers

**DOI:** 10.3390/genes17050559

**Published:** 2026-05-08

**Authors:** Ebru Dolu, Savaş Orhan, Celal Bulgay, Hasan Hüseyin Kazan, David Varillas-Delgado, Attila Szabo, Mehmet Ali Ergün, Luca Paolo Ardigò

**Affiliations:** 1 Institute of Health Sciences, Bingöl University, Bingöl 12000, Türkiye; ebrudolu2123@gmail.com (E.D.); sirac.2185@gmail.com (S.O.); 2 Sports Science Faculty, Bingöl University, Bingöl 12000, Türkiye; 3 Department of Medical Biology, Gulhane Faculty of Medicine, University of Health Sciences, Ankara 06010, Türkiye; hasanhuseyin.kazan@sbu.edu.tr; 4 Exercise and Sport Science, Faculty of Health Sciences, Universidad Francisco de Vitoria, 28223 Pozuelo, Spain; david.varillas@ufv.es; 5 Faculty of Health and Sport Sciences, Széchenyi István University, H-9026 Győr, Hungary; szabo.attila@sze.hu; 6 Department of Medical Genetics, Faculty of Medicine, Gaz University, Ankara 06560, Türkiye; aliergun@gazi.edu.tr; 7 Department of Teacher Education, NLA University College, 0166 Oslo, Norway

**Keywords:** athletes, wrestlers, handgrip strength, *ACTN3*, *BDNF*

## Abstract

Background/Objectives: Handgrip strength (HGS) is a widely used indicator of upper-limb muscular strength and a practical proxy for neuromuscular performance across both clinical and athletic contexts. Although HGS is heritable, evidence supporting specific genetic contributors in elite athletes remains limited. Thus, the present study investigated the associations of two functional polymorphisms, *BDNF* rs6265 (p.Val66Met) and *ACTN3* rs1815739 (p.R577X), with HGS performance in elite wrestlers, integrating neuromuscular and muscle fiber-related biological pathways. Methods: The present study included 613 subjects (56 elite male wrestlers (mean age: 22.35 ± 5.34 years; training experience: 13.40 ± 3.85 years) and 557 healthy individuals drawn from a public database). HGS measurements were performed using a digital hand dynamometer. Results: Genotyping was performed on DNA extracted from peripheral blood using a high-density single nucleotide polymorphism (SNP) array. Neither *BDNF* rs6265 nor *ACTN3* rs1815739 was significantly associated with HGS in elite wrestlers (*p* > 0.05), and effect estimates were negligible. In addition, *ACTN3* rs1815739 genotype and allele frequencies were comparable between wrestlers and the reference population, indicating no enrichment of this variant in the elite cohort. In this sample of elite male wrestlers, *BDNF* rs6265 and *ACTN3* rs1815739 were not associated with HGS, and *ACTN3* rs1815739 was not enriched relative to a national reference population. Conclusions: These findings suggest no detectable effects of single candidate variants on HGS under the current study design in highly trained athletes; however, this interpretation should be made cautiously given cohort-specific limitations and does not preclude their potential contribution within the broader polygenic architecture of strength-related traits. Future research employing larger, well-powered, and multi-cohort designs and polygenic approaches is warranted to further elucidate the genetic basis of strength phenotypes.

## 1. Introduction

Handgrip strength (HGS) is a commonly used field test for assessing upper-extremity muscular strength because of its simplicity, high test–retest reliability, and minimal equipment requirements [1,2,3,4,5,6]. A substantial body of research across different ages, sexes, and sports disciplines has established HGS as a reliable indicator for monitoring and evaluating athletic performance [7,8,9,10,11,12]. Additionally, several studies have reported positive associations between HGS and key performance components, including snatch performance, lower-limb strength, isokinetic muscle force, and aerobic capacity [13,14,15,16,17,18]. However, its relevance as a performance indicator may vary across different sports disciplines [19,20]. Therefore, it should be considered as a practical, but context-dependent, measure of muscular strength.

Beyond its relevance to athletic performance, handgrip strength (HGS) is increasingly recognized as a practical biomarker associated with various health-related outcomes, including depression, metabolic disorders, type 2 diabetes, cardiovascular function, sarcopenia, cognitive performance, and mental health, as evidenced by clinical and epidemiological research [4,6,10,21,22,23,24,25,26,27,28]. However, these associations should be interpreted with caution, as HGS represents a limited indicator of overall physical function rather than a definitive biomarker of general health.

A growing body of evidence suggests that muscular strength is strongly influenced by genetic factors [29,30]. Nevertheless, heritability estimates for strength-related phenotypes, including HGS, vary widely (30–82%), likely reflecting differences in study populations, measurement protocols, and analytical approaches. Twin studies, which offer more controlled designs for disentangling genetic and environmental influences, generally report more consistent estimates of approximately 50–60%. Under more specific and controlled testing conditions (e.g., isometric, isotonic, and isokinetic muscle actions), genetic factors may account for an even greater proportion of variance, approaching ~85%, suggesting that heritability estimates are highly context-dependent [31,32,33,34,35,36,37].

Genome-wide association studies (GWAS) have identified multiple single-nucleotide polymorphisms (SNPs) associated with HGS, many of which are located within or near genes implicated in skeletal muscle structure, neuromuscular function, and tissue remodeling [37]. Importantly, several of these variants have been reported in elite strength athletes, suggesting that molecular pathways underlying HGS in the general population may also contribute to exceptional performance in strength-oriented sports [38,39]. Beyond the GWAS approach, focusing on muscular strength-related loci in terms of genetic associations in a large cohort would be informative.

Wrestling is a high-intensity combat sport that demands exceptional gripping ability, explosive strength, neuromuscular coordination, and technical proficiency [40]. Within this context, the α-actinin-3 (*ACTN3*) and brain-derived neurotrophic factor (*BDNF*) genes were selected as candidate genes due to their established roles in strength and performance-related mechanisms. The α-actinin-3 gene encoded by the *ACTN3* gene has been consistently associated with muscle strength and power performance owing to its expression in fast-twitch (type II) muscle fibers. In particular, the *ACTN3* c.1729C>T polymorphism (rs1815739) influences α-actinin-3 expression, with the CC (RR) genotype linked to enhanced power output and the TT genotype associated with reduced high-velocity force production [41,42,43]. In contrast, the BDNF protein is implicated in neuromuscular adaptation, motor learning, and neural regulation of strength. The *BDNF* c.196G>A polymorphism (rs6265) affects activity-dependent BDNF secretion and plays a key role in synaptic plasticity and motor learning processes that may influence skill acquisition and neural components of strength expression [44,45,46,47]. Together, these genes represent complementary structural and neural mechanisms underlying strength-related performance, providing a biologically grounded rationale for their investigation in wrestling.

Despite the well-established role of genetic factors in strength-related phenotypes, research examining associations between HGS and genetic variation in elite athletic populations remains limited, particularly regarding the population-specific cohorts, the number of investigated genetic markers, and the integration of relevant performance measures. Therefore, the aim of the present study was to investigate the association between HGS and the polymorphisms *ACTN3* rs1815739 and *BDNF* rs6265 in elite wrestlers. Clarifying the genetic basis of HGS in wrestlers may help contextualize inter-individual differences and guide future work on performance-related genetic profiles and individualized training approaches.

## 2. Materials and Methods

### 2.1. Ethical Approval

The present study was approved by the Clinical Research Ethics Committee of Gazi University (Approval no: 642, 2023). Both written and verbal consent was obtained from all participants prior to data collection. The study was carried out in compliance with the ethical principles outlined in the Declaration of Helsinki [48] and followed the Strengthening the Reporting of Genetic Association Studies (STREGA) guidelines, which extend the Strengthening the Reporting of Observational Studies in Epidemiology (STROBE) recommendations [49].

### 2.2. Study Design

The present study was designed as a cross-sectional, observational genotype–phenotype association study. The primary objective was to examine the relationship between functional genetic polymorphisms, *ACTN3* (rs1815739) and *BDNF* (rs6265), and maximal HGS in a homogeneous cohort of elite male wrestlers. All phenotypic HGS and genotypic data were collected at a single time point, without carrying out any experimental intervention or longitudinal follow-up. As previously mentioned, this study followed the STREGA recommendations for reporting genetic association studies and adhered to established methodological standards for observational research in sports genomics. Comparisons of genotype and allele frequencies were additionally performed using reference data from a national genomic database to provide population-level context. Further details are reported in the following sections.

### 2.3. Participants

A total of 56 elite male wrestlers affiliated with the Turkish Wrestling Federation and actively competing in international championships were enrolled in the present study. The participants had a mean age of 22.35 ± 5.34 years, a mean height of 172.24 ± 6.15 cm, a mean body weight of 80.65 ± 15.70 kg, and a mean training experience of 13.40 ± 3.85 years. The mean HGS value was 50.81 ± 7.05 kg. For documentative purposes, genotyping data from 557 healthy individuals obtained from the Turkish Genome Project (TGP) database (https://tgd.tuseb.gov.tr/; accessed on 3 December 2025) were used as the control group. As no phenotype data were available for this group, the comparison is limited to allele and genotype frequencies and does not represent a case–control association. Therefore, no performance-related inferences can be drawn. All participants were of Turkish nationality and of Caucasian origin.

### 2.4. Handgrip Strength Measurement

HGS was assessed to evaluate upper-extremity muscle strength using a standard Smedley-type hand dynamometer (Grip-D 5101; Takei, Niigata, Japan), following a standardized testing protocol with participants in a standing position. The dynamometer was calibrated according to the manufacturer’s guidelines before each testing session and periodic checks were performed using known weights to ensure measurement accuracy and reliability. The reliability and validity of handgrip strength measurements using Smedley-type dynamometers have been well-established in previous studies [2,3,12,50,51].

Prior to testing, participants completed a standardized warm-up protocol. All measurements were conducted between 15:00 and 17:00 o’clock to minimize diurnal variation. During the test, wrestlers were instructed to hold the dynamometer in their dominant hand, fully extend the arm downward, and maintain an approximately 15° angle between the upper arm and the trunk to ensure consistency across trials.

The grip span was adjusted so that the second interphalangeal joints of the fingers aligned with the handle while the thumb rested along the base of the palm. Participants exerted maximal isometric force for approximately five seconds. The dominant hand was tested twice (~60 s rest interval), and the highest value was recorded to the nearest 0.1 kg. No standardized verbal encouragement was provided during the testing. Participants were only instructed to begin when they felt ready [2,12]. All measurements were performed under the same conditions and supervised by an experienced researcher.

### 2.5. Genotyping

Genomic DNA was extracted from peripheral blood samples using the QIAmp Blood Mini Kit (Qiagen, Steinhausen, Switzerland) following the manufacturer’s protocols. Genotyping was performed using the Axiom™ Precision Medicine Diversity Array Kit (Thermo Scientific, Waltham, MA, USA) according to the supplier’s protocol. After genotyping, raw data was quality checked, and genotype call rates were selected as 0.95. Subsequently, as a part of the ongoing project, genotypes for the *ACTN3* rs1815739 and *BDNF* rs6265 polymorphisms were curated and processed for statistical association analysis.

Finally, the effects and/or associations of selected polymorphisms on/with the expression of any genes in the tissues which may affect muscle strength were checked and obtained from GTEx Portal (https://www.gtexportal.org/; dbGaP Accession phs000424.v10.p2; accessed on 8 March 2026).

### 2.6. Statistical Analysis

In the present study, an a priori power analysis was conducted using G*Power 3.1 software to determine the required sample size. According to Cohen’s [52] criteria, effect sizes were classified as 0.10 (small), 0.30 (medium), and 0.50 (large). A large effect size (0.50) was assumed. The power analysis was performed using the chi-square (χ^2^) test family (goodness-of-fit tests: contingency tables) with a statistical power of 83%. The analysis indicated that a minimum sample size of 55 wrestlers was required.

Statistical analyses were performed using Microsoft Excel and SPSS version 29.0 for Mac. Data integrity was verified prior to analysis, and normality was assessed using the Shapiro–Wilk test, confirming that all variables were normally distributed. The differences in HGS among genotypes were assessed using analysis of covariance (ANCOVA), with genotype as a fixed factor and age, height, body weight, and sports experience included as covariates due to their potential influence on strength performance. Post hoc comparisons were conducted using the Bonferroni correction where appropriate.

Genotypic and allelic frequencies were calculated, and the Hardy–Weinberg equilibrium (HWE) was assessed using the chi-square (χ^2^) test or Fisher’s exact test, as appropriate (*p* > 0.05). Effect sizes were calculated as partial eta squared (η^2^) for ANCOVA models and interpreted as trivial (<0.01), small (0.01–0.06), medium (0.06–0.14), and large (>0.14) [53]. Significance was set at *p* < 0.05, and all tests were two-sided. Effect estimates are reported with 95% confidence intervals (CI).

## 3. Results

**Figure 1:**
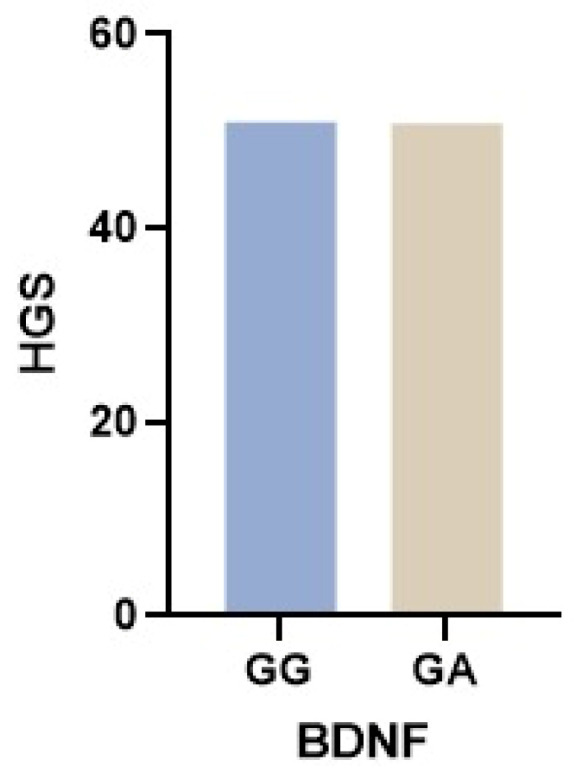
No statistically significant differences between *BDNF* and HGS, *p* > 0.05.

For the *BDNF* rs6265 polymorphism, no statistically significant differences in HGS were observed between genotype groups (F = 0.197, *p* = 0.65, partial η^2^ = 0.004; Table 1; Figure 1).

**Figure 2:**
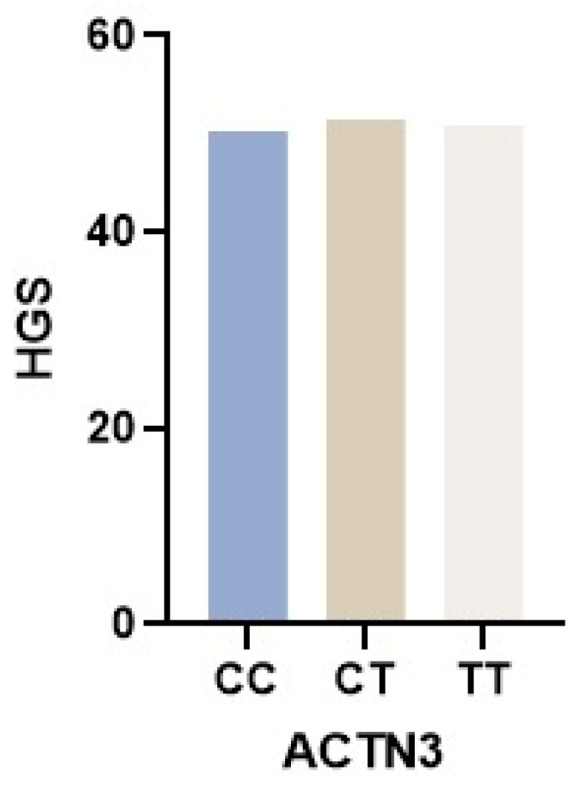
No statistically significant differences between *ACTN3* and HGS, *p* > 0.05.

Similarly, no statistically significant differences in HGS were observed between genotype groups for the *ACTN3* rs1815739 polymorphism (F = 0.005, *p* = 0.99, partial η^2^ = 0.001; Table 2; Figure 2).

To contextualize these findings at the population level, allele and genotype frequencies were documented using data from 557 healthy individuals obtained from the TGP database. While information for *BDNF* rs6265 was unavailable, the distribution of *ACTN3* rs1815739 genotypes in wrestlers closely resembled that of the reference population. Specifically, the proportion of homozygotes (26.79%) and the frequency of the T allele (0.46) in wrestlers were comparable to those reported in the database (22.80% homozygotes; T allele frequency = 0.47).

Finally, we checked the role of selected polymorphisms on the expression of any genes in the GTEx portal. Accordingly, both polymorphisms have been linked to altered expression of diverse genes. Importantly, rs1815739 was associated with down-regulation of the *ACTN3* and cathepsin F (*CTSF*) genes in skeletal muscle and adipose tissue, whereas it was associated with up-regulation of the Bardet-Biedl syndrome 1 (*BBS1*) gene in those tissues. Although not in muscle or adipose tissues, rs6265 was linked to the down-regulation of *BDNF* in the heart and BDNF antisense RNA (a long non-coding RNA; *BDNF-AS*) in the brain, while it was associated with up-regulation of Lin-7 cell polarity scaffold C (*LIN7C*) in arteries (Table 3).

## 4. Discussion

The present study evaluated whether *BDNF* rs6265 and/or *ACTN3* rs1815739 polymorphisms were associated with HGS in elite wrestlers. After adjustment for age, height, body weight, and training experience, no statistically significant genotype–phenotype associations were detected, and effect estimates were small. Accordingly, no evidence of an independent contribution of either variant to maximal HGS was observed under the present conditions in this elite cohort.

This null finding should be interpreted considering the phenotype definition. HGS is a practical and widely used proxy of muscular strength; however, it represents a single, static measure and may not adequately capture the neuromuscular and power-related characteristics that are more plausibly linked to *ACTN3* and *BDNF*-related biology (e.g., rapid force development, explosive/dynamic strength, and task-specific motor control) in elite wrestlers.

In highly trained athletes, long-term sport-specific training and neuromuscular adaptations can compress between-individual variability in single field tests [53,54,55]. Such restricted variability may reduce statistical sensitivity and make it particularly difficult to detect the small effects expected for common variants, even when they are biologically relevant [56,57]. In addition, although an a priori power analysis was conducted, the assumption of a large effect size may not be appropriate for genetic studies. Given that the effects on complex traits containing HGS are typically small, the current sample may be insufficient to detect such effects. Therefore, the absence of significant findings may partly be explained by limited statistical power.

In the present study, the maximum value obtained from the dominant hand was used as an index of peak voluntary force, consistent with common reporting practice. However, wrestling training is likely to induce bilateral adaptations, and the absence of non-dominant-hand data may have reduced the sensitivity to detect genotype-related differences or asymmetry patterns. Future studies should incorporate bilateral HGS (dominant and non-dominant values, mean/maximum measures, and asymmetry indices) to better characterize strength expression in grappling athletes.

To contextualize the present findings, allele frequencies were reported using data from 557 healthy individuals in the TGP database. Genotype information for rs6265 was not available in this dataset. For rs1815739, 127 individuals (22.80%) were identified as homozygous (TT), and the T-allele frequency was reported as 0.47. In our cohort, the proportion of TT homozygotes (26.79%) and the T-allele frequency (0.46) were found to be similar to those observed in the reference population. However, no formal statistical comparison (e.g., chi-square test) was performed. Accordingly, no evidence of enrichment of rs1815739 in elite wrestlers was observed. It should also be noted that this comparison was limited to allele frequencies, as phenotype data were not available for the reference group; therefore, these findings should be interpreted with caution.

Overall, the present findings are consistent with the view that HGS and related strength traits are highly polygenic, with variation arising from many loci of small effect alongside substantial environmental and training influences [37,38,58,59,60,61]. In elite cohorts, the contribution of any single common variant is expected to be particularly difficult to detect against the background of extensive training adaptation and other unmeasured influences, including regulatory and epigenetic mechanisms [61]. In this context, well-described negative findings are informative and highlight the limited explanatory value of single-SNP candidate approaches for complex performance phenotypes in highly trained athletes.

Within this polygenic framework, *BDNF* rs6265 and *ACTN3* rs1815739 remain biologically plausible candidates. *BDNF* rs6265 influences activity-dependent secretion of BDNF protein and has been linked to pathways relevant to synaptic plasticity, motor learning, cortical excitability, and neuromuscular coordination [47]. *ACTN3* rs1815739 determines α-actinin-3 expression in fast-twitch fibers, which has been related to rapid force production and resistance to eccentric fatigue in broader populations [43].

The present findings from expression prediction using the GTEx portal also underlined that those polymorphisms were linked to down- or up-regulation of *ACTN3*, *BBS1*, *CTCF*, *BDNF*, *LIN7C* and *BDNF*-*AS* in the skeletal muscle, adipose tissue, heart, artery and brain which may affect the HGS capability by regulating muscle coordination and/or neurological concentration. Nonetheless, even for functional variants, effects on performance measures can be modest and may further be attenuated in elite athletes due to long-term training adaptations and compensatory mechanisms. In this context, using HGS as the primary phenotype constitutes a limitation, as its static, single-joint nature may not capture genotype-related differences. Given that *ACTN3* and *BDNF* are more closely linked to explosive strength, rate of force development, and neuromuscular coordination, HGS may lack sufficient sensitivity to detect these genetic effects in elite athletes.

The present study is strengthened by its exclusive focus on elite wrestlers, which reduces heterogeneity in training status and improves internal validity, and by the evaluation of two well-characterized functional polymorphisms. Several limitations should also be considered: (i) the modest sample size may limit statistical power to detect small genetic effects, (ii) restriction to a single sport restricts the generalizability, (iii) reliance on HGS as the sole performance metric may not capture the multidimensional neuromuscular traits relevant to these variants, (iv) the inclusion of only male wrestlers restricts the generalizability of the findings to female wrestlers, (v) the lack of consideration of sport-specific variables such as weight category, wrestling style, competition level, and training phase would weakens the cohort characteristics, and (vi) epigenetic and/or regulatory factors that may contribute to regulation of gene expression were not assessed.

## 5. Conclusions

The present study indicates that no detectable independent effect of *ACTN3* rs1815739 or *BDNF* rs6265 on maximal HGS was observed under the current study design in this cohort of elite wrestlers. These findings should be interpreted with caution, as they are limited to the present sample and phenotype definition and do not preclude potential contributions of these variants within the broader polygenic architecture of strength-related traits.

Considering the polygenic architecture of performance-related traits, future studies should emphasize larger, multi-centered, and more diverse cohorts, alongside more comprehensive and functionally relevant phenotyping (e.g., explosive strength, rate of force development, anaerobic capacity, and muscle architecture). Moreover, the integration of multi-variant and advanced analytical approaches such as GWAS-informed models, polygenic risk scores, and combined genetic–epigenetic frameworks will be crucial for achieving a more nuanced and robust understanding of the genetic determinants of strength-related performance.

## Figures and Tables

**Table 1:** Association analysis of the *BDNF* rs6265 genotypes with HGS performance.

Variable	Genotype	*N*	M ± SD	F	*p*	η^2^	95% CI
HGS (kg)	GG	37	50.98 ± 7.13	0.197	0.65	0.004	48.25–52.98
	GA	19	50.84 ± 7.09				48.19–54.91
	AA	---	---				---

Values expressed as M ± SD adjusted for age, height, body weight, and sport experience by ANCOVA; M: mean; SD: standard deviation; (η^2^): partial eta squared.

**Table 2:** Association analysis of the *ACTN3* rs1815739 genotypes with HGS performance.

Variable	Genotype	*N*	M ± SD	F	*p*	η^2^	95% CI
HGS (kg)	CC	19	50.37 ± 6.35	0.005	0.99	0.001	47.54–54.20
	CT	22	51.46 ± 8.21				47.92–54.20
	TT	15	50.86 ± 6.46				47.11–54.57

Values expressed as M ± SD adjusted for age, height, body weight and sport experience by ANCOVA; M: mean; SD: standard deviation; (η^2^): partial eta squared.

**Table 3:** Association of selected polymorphisms with expression of different genes in diverse tissues using expression Quantitative Trait Locus (eQTL).

Polymorphism	Associated Gene	Tissue	*p*-Value	NES
rs1815739	*ACTN3*	Skeletal muscle	3.7 × 10^−158^	−0.73
		Adipose	2.2 × 10^−32^	−0.55
	*BBS1*	Adipose	9.5 × 10^−14^	0.16
		Skeletal muscle	4.7 × 10^−10^	0.16
	*CTSF*	Adipose	5.2 × 10^−13^	−0.14
		Skeletal muscle	4.0 × 10^−7^	−0.11
rs6265	*BDNF*	Heart	0.00015	−0.17
	*LIN7C*	Artery	4.2 × 10^−8^	0.17
	*BDNF-AS*	Brain	0.0000019	−0.20

NES: normalized effect size.

## Data Availability

Data are available for research purposes upon reasonable request to the corresponding authors.
